# Dietary sugars modulate bacterial-fungal interactions in saliva and inter-kingdom biofilm formation on apatitic surface

**DOI:** 10.3389/fcimb.2022.993640

**Published:** 2022-11-09

**Authors:** Thais de Cássia Negrini, Zhi Ren, Yilan Miao, Dongyeop Kim, Áurea Simon-Soro, Yuan Liu, Hyun Koo, Rodrigo Alex Arthur

**Affiliations:** ^1^ Biofilm Research Laboratories, Center for Innovation & Precision Dentistry, School of Dental Medicine, University of Pennsylvania, Philadelphia, PA, United States; ^2^ Department of Orthodontics and Divisions of Pediatric Dentistry & Community Oral Health, School of Dental Medicine, University of Pennsylvania, Philadelphia, PA, United States; ^3^ Department of Clinical Analysis, School of Pharmaceutical Sciences, Sao Paulo State University, Araraquara, Brazil; ^4^ Department of Preventive Dentistry, School of Dentistry, and Institute of Oral Bioscience, Jeonbuk National University, Jeonju, South Korea; ^5^ Department of Stomatology, School of Dentistry, University of Seville, Seville, Spain; ^6^ Preventive & Restorative Sciences, School of Dental Medicine, University of Pennsylvania, Philadelphia, PA, United States; ^7^ Department of Preventive and Community Dentistry, Dental School, Federal University of Rio Grande do Sul, Porto Alegre, Brazil

**Keywords:** *S. mutans*, *C. albicans*, saliva, sucrose, inter-kingdom aggregate, EPS

## Abstract

Bacteria and fungi can interact to form inter-kingdom biofilms in the oral cavity. *Streptococcus mutans* and *Candida albicans* are frequently detected in saliva and in dental biofilms associated with early childhood caries (tooth-decay), a prevalent oral disease induced by dietary sugars. However, how different sugars influence this bacterial-fungal interaction remains unclear. Here, we investigate whether specific sugars affect the inter-kingdom interaction in saliva and subsequent biofilm formation on tooth-mimetic surfaces. The microbes were incubated in saliva containing common dietary sugars (glucose and fructose, sucrose, starch, and combinations) and analyzed *via* fluorescence imaging and quantitative computational analyses. The bacterial and fungal cells in saliva were then transferred to hydroxyapatite discs (tooth mimic) to allow microbial binding and biofilm development. We found diverse bacterial-fungal aggregates which varied in size, structure, and spatial organization depending on the type of sugars. Sucrose and starch+sucrose induced the formation of large mixed-species aggregates characterized by bacterial clusters co-bound with fungal cells, whereas mostly single-cells were found in the absence of sugar or in the presence of glucose and fructose. Notably, both colonization and further growth on the apatitic surface were dependent on sugar-mediated aggregation, leading to biofilms with distinctive spatial organizations and 3D architectures. Starch+sucrose and sucrose-mediated aggregates developed into large and highly acidogenic biofilms with complex network of bacterial and fungal cells (yeast and hyphae) surrounded by an intricate matrix of extracellular glucans. In contrast, biofilms originated from glucose and fructose-mediated consortia (or without sugar) were sparsely distributed on the surface without structural integration, growing predominantly as individual species with reduced acidogenicity. These findings reveal the impact of dietary sugars on inter-kingdom interactions in saliva and how they mediate biofilm formation with distinctive structural organization and varying acidogenicity implicated with human tooth-decay.

## Introduction

Early childhood caries (tooth-decay) is a biofilm-dependent and diet-modulated disease that affects more than 600 million children globally (Uribe et al., 2021). Rapidly-fermentable sugar-rich food plays an essential role on the etiology of this disease (Sheiham and James, 2015; Marshall, 2019), since it promotes biofilm accumulation and acid production that cause mineral loss and the onset of dental caries (Dawes, 2003). Within the highly diverse microbial community found in the oral cavity, bacterial-fungal (inter-kingdom) interactions have been increasingly recognized in the disease pathogenesis and are associated with enhanced dental biofilm virulence (Negrini et al., 2019). *Candida albicans* (a fungus) is frequently co-detected with high numbers of *Streptococcus mutans* (a cariogenic bacterium) in biofilms formed on tooth surfaces of toddlers with severe childhood caries (Koo and Bowen, 2014; Hajishengallis et al., 2017; Xiao et al., 2018). Notably, a high carriage of both *S. mutans* and *C. albicans* were found in the saliva of the diseased children (Xiao et al., 2018), who are frequently exposed to dietary sugars. Whether the sugars can influence the early interaction of these two microbes in saliva to modulate the subsequent biofilm formation remains unclear.

Among the dietary sugars, sucrose is the most cariogenic since it enhances both dental biofilm accumulation and its virulence (Bowen et al., 2018). This sugar is a substrate for the synthesis of extracellular polymers (EPS), termed glucans, by glucosyltransferases (Gtfs) secreted by *S. mutans*. EPS glucans enhance microbial adhesion to the tooth surface and promote biofilm accumulation, while enmeshing the microorganisms to provide cohesion, protection, and altered diffusion properties that increases cariogenicity (Bowen and Koo, 2011; Kim et al., 2018). Conversely, sucrose can be also metabolized into acids lowering the biofilm pH that promotes enamel demineralization (Bowen et al., 2018). Based on these deleterious effects and dietary sugars as risk factor for dental caries, the World Health Organization has recommended that the high energy intake be provided by starch instead of the consumption of free-sugar (such as sucrose) containing foods (WHO, 2015). However, in addition to lack of evidence showing association between starch consumption and reduced caries risk, starchy foods are notoriously retentive in the oral cavity which appears to enhance cariogenic potential especially when consumed simultaneously or interspersed with sucrose (Halvorsrud et al., 2019). Available evidence shows that the combination of sucrose and starch boosts biofilm formation, enhances EPS and acid production, and increases the biofilm cariogenic potential (Ribeiro et al., 2005; Duarte et al., 2008; Klein et al., 2010; Botelho et al., 2016).

A critical step of dental biofilm development is the initial attachment of microbial cells present in saliva onto the tooth surface. Human saliva also harbors microbial aggregates in addition to free-living single-cells (Koop et al., 1989; Cárlen et al., 1996; Simon-Soro et al., 2022). Interestingly, *C. albicans* on its own does not colonize or form biofilms on tooth-mimetic surfaces effectively (Falsetta et al., 2014), but under sucrose-rich condition of early childhood caries, this fungal organism forms abundant inter-kingdom biofilms with *S. mutans* primarily due to symbiotic sugar utilization that produces elevated amounts of EPS and acids (Gregoire et al., 2011; Falsetta et al., 2014; Hwang et al., 2017; Kim et al., 2021). Given that *S. mutans* and *C. albicans* are found in high levels in the saliva of children with early childhood caries (Xiao et al., 2018; Kirthiga et al., 2019), a question arises on whether they interact in saliva in the presence of sugars, modulate surface colonization, and influence biofilm initiation.

Here, we investigate how different dietary sugars affect the inter-kingdom interactions in saliva and subsequent biofilm formation on tooth-mimetic surfaces. We hypothesize that specific sugars induce *C. albicans* and *S. mutans* co-aggregation in saliva which can modulate surface colonization and biofilm development. Using a saliva-based biofilm model, multiscale fluorescence imaging, and quantitative computational analyses, we find diverse bacterial-fungal co-aggregates in saliva which vary in size, structure, and spatial organization depending on the type of sugars. Sucrose and starch in combination with sucrose induce the formation of large and organized bacterial-fungal aggregates characterized by bacterial clusters co-bound with fungal cells enmeshed in extracellular glucan matrix. Notably, these inter-kingdom aggregates could attach to the tooth-mimetic surfaces and grow into highly structured biofilms with greater biomass and acidogenicity than biofilms originated from microbes in saliva without sugar or with glucose and fructose. Our results reveal that sucrose and its association with starch promote the accumulation of virulent biofilms by inducing early inter-kingdom aggregation in saliva that develops into enlarged biofilm structures with enhanced acidogenesis implicated with severe early childhood caries.

## Materials and methods

### Ethics statement

This study was reviewed and approved by the Institutional Review Board of University of Pennsylvania (IRB #824243). The saliva donors provided their written informed consent to participate in this study

### *In vitro* co-aggregation, binding, and biofilm model

*S. mutans* UA 159 serotype c (an established cariogenic dental pathogen and well-characterized EPS producer) and *C. albicans* SC5314 (a well-characterized fungal strain) were used to generate inter-kingdom aggregates and biofilms. They were grown in ultrafiltered (10-kDa molecular-mass cutoff membrane; Millipore, MA, USA) tryptone-yeast extract broth (UFTYE; 2.5% tryptone and 1.5% yeast extract at pH 7.1 for *S. mutans* and at pH 5.5 for *C. albicans*) with 1% (wt/vol) glucose at 37°C and 5% CO_2_ until optical density at 600 nm of 1.0 (~10^9^ CFU of *S. mutans*/mL) and 0.8 (~10^6^ CFU of *C. albicans*/mL), respectively. Filter-sterilized clarified human whole saliva (CLS) was collected from healthy donors (amylase activity = 104.1 ± 18.4 U/mL), prepared, and pooled as previously described (Koo et al., 2000). Planktonic bacterial and fungal cells were mixed in CLS supplemented with the following dietary sugars: 1) glucose 0.5% (wt/vol) + fructose 0.5%; 2) starch 1% (soluble starch [80% amylopectin and 20% amylose, Sigma Chemical Company, St. Louis, MO); 3) starch 1% + glucose 0.5% + fructose 0.5%; 4) sucrose 1% and 5) starch 1% + sucrose 1%. The resulted inoculum contained 10^8^ CFU/mL *S. mutans* and 10^6^ CFU/mL *C. albicans* (predominantly yeast form). CLS without sugar was used as control. Suspensions were kept under rocking at 37°C for 60 minutes to allow microbial co-aggregation. Suspensions were then transferred to 24-well plates (2.8 mL per well) and aggregates were allowed to bind to vertically suspended saliva-coated hydroxyapatite discs (sHA; tooth mimic with a surface area 2.7 ± 0.2 cm^2^; Clarkson Chromatography Products, Inc., South Williamsport, PA) (Falsetta et al., 2014) under constant rocking for 60 minutes. sHA discs were then transferred to new 24-well plates containing UFTYE pH 7.1 supplemented with the same sugar or their combinations as described above. UFTYE without sugar was used as control. Biofilms were grown at 37°C under 5% CO_2_ for 19 hours. The following samples were collected and examined using confocal laser scanning microscopy (CLSM) combined with quantitative computational image analysis: 1) microbial suspensions in CLS after 60-min incubation; 2) sHA disks after the 60-min microbial binding; and 3) 19h biofilms formed. Biofilms were also assessed by microbiological assays to determine the counts of viable bacterial and fungal cells (CFU) and total insoluble dry-weight.

### Qualitative and quantitative CLSM analysis

To visualize the bacterial/fungal cells and EPS within the aggregates in CLS, Alexa Fluor 647-dextran conjugate (647/668 nm; Molecular Probes Inc.) was added in the CLS during the co-aggregation to label EPS formation. For biofilms, Alexa Fluor 647-dextran conjugate was added in UFTYE in each well during the biofilm growth (Xiao et al., 2012; Falsetta et al., 2014). After the 60-min co-aggregation, *S. mutans* was stained with Syto 9 green-fluorescent nucleic acid stain (485/498 nm; Molecular Probes Inc., Eugene, OR, USA) and *C. albicans* was stained with concanavalin A (ConA) lectin conjugated with tetramethylrhodamine (555/580 nm; Molecular Probes Inc., Eugene, OR, USA) (Falsetta et al., 2014). Immediately before imaging, aliquots of CLS microbial suspension was pipetted onto pre-solidified agarose (1%) on a glass slide followed by a cover slip to immobilize the microbes while preserving their structure. This method did not cause aggregation of planktonic microorganisms either alone or mixed. High-resolution confocal microscopy was performed using a 40 × water immersion objective (numerical aperture = 1.2) on a Zeiss LSM 800 confocal microscope (Zeiss, Germany). For imaging of surface-bound microbial structures or biofilms, sHA discs were stained with Syto 9 and then with ConA to label bacterial and fungal cells, respectively. Confocal imaging was performed using a 20 × water immersion objective (numerical aperture = 1.0) on the LSM 800 confocal system. Each field of view was sequentially imaged as follows: 1) Syto 9 channel (excitation with 488 nm and collection by 480/40 nm emission filter), 2) ConA channel (excitation with 561 nm and collection by 560/40 nm emission filter and 3) Alexa Fluor 647 channel (excitation with 640 and collection by 670/40 nm emission filter). Biofilm samples were imaged using a 20 × LPlan (numerical aperture = 1.05) water immersion objective on a Leica SP8 multi-photon microscope (Leica Microsystems, Buffalo Grove, IL, USA).

For computational image analyses, the surface area (µm^2^) of individual aggregates was determined for each of the tested conditions using ImageJ software. Briefly, images were individually thresholded and converted into binary data and aggregate surface area was calculated using 2 µm^2^ as the minimum particle size. Further computational analyses on biofilms were performed using COMSTAT with customized scripts for MATLAB software (Mathworks, Natick, MA, USA) to calculate the biovolume as previously described (Xiao et al., 2012). The biovolume occupied by bacterial cells, fungal cells or EPS respectively within intact biofilms was calculated. Amira 5.4.1 software (Visage Imaging, San Diego, CA, USA) was used to create 3D renderings to visualize the overall architecture of the biofilms. Three independent experiments were done for each tested condition.

### Growth kinetics of surface-bound aggregates

To investigate how surface-bound aggregates grew spatiotemporally into biofilms, we employed time-lapse live imaging coupled with computational image analysis (Paula et al., 2020; Kim et al., 2021). Briefly, *S. mutans* UA 159 and *C. albicans* SN250 tagged with red fluorescent protein (tdTomato; gift from Damian J. Krysan, University of Iowa) were used to generate surface-bound aggregates as described above. The sHA disc harboring bound cells was then pre-stained with Syto 9 for 10 minutes to fluorescently label the bacterial cells. The disc was aseptically transferred into a flow-cell microfluidic device (FC310, BioSurface Technologies, Bozeman, MT, USA) for real-time confocal imaging. UFTYE supplemented with 1% sucrose and with Syto 9 was continuously provided at a constant flow rate (100 μL/min) for up to 600 minutes to allow continuous bacterial cell labelling during the biofilm growth. Time-lapsed images were captured every 30 minutes using Zeiss LSM 800 confocal microscope (Zeiss, Germany) equipped with 10 × objective (numerical aperture = 0.4). A Gaussian filter (radius = 0.8) in ImageJ was applied to the denoise the images which were then thresholded/binarized using the Otsu method in the same software. Two areas of interest containing bacterial-fungal aggregates or bacteria alone, respectively were tracked over time. The Raw Integrated Density of the bacterial channel at each time point was determined to generate the growth curves of the bacterial cells.

### Biochemical and microbiological analyses

A separate set of biofilms was used for standard microbiological analysis. Briefly, the biofilm was collected from sHA discs and homogenized *via* water bath sonication followed by probe sonication (30 s pulse at an output of 7 W; Branson Sonifier 150, Branson Ultrasonics, Danbury, CT, USA) as described previously (Xiao et al., 2012; Falsetta et al., 2014). An aliquot of biofilm suspension was centrifuged, the pellet was washed twice with milli-Q water, dried in an oven and weighed to determine the total insoluble dry-weight (mg) of biofilms (Koo et al., 2003). Moreover, the homogenized biofilm suspension was also diluted and plated on tryptic-soy agar supplemented with 5% sheep blood to determine the total number of viable cells (CFU) of *S. mutans* and *C. albicans*, respectively. Three independent experiments were conducted for each tested condition.

### Acidogenic profile of biofilms

The acidogenic profile of biofilms was assessed by pH drop assays. We first performed a standard pH drop through fermentation of glucose. Briefly, 19h biofilms on HA disks were incubated with 20 mM potassium phosphate buffer pH 7.2 (supplemented with 1 mM MgCl_2_ and 50 mM KCl) for 1 hour at 37°C and 5% CO_2_. In each experimental setup, two HA disks with pre-formed biofilms were then incubated in 6 mL of salt solution (50 mM KCl plus 1 mM MgCl_2_, pH 7.0) containing glucose at a final concentration of 1%. The pH was continuously measured using a glass electrode every 10 min over a period of 120 min (Belli et al., 1995; Jeon et al., 2011). Additionally, in a separate set of biofilms the culture medium was replaced at 19h by fresh medium supplemented with the same dietary sugars used in each group: 1) glucose 0.5% (wt/vol) + fructose 0.5%; 2) starch 1%; 3) starch 1% + glucose 0.5% + fructose 0.5%; 4) sucrose 1% or 5) starch 1% + sucrose 1%. Aliquots (0.1 mL) of the culture medium were taken at specific time points (2, 4, 6, 8 and 10h after medium replacement), and pH values were measured using a glass electrode (Futura Micro Combination pH electrode, 5 mm diameter, Beckman Coulter Inc., CA). Data were presented as amount of pH drop and hydrogenionic concentration. Three independent experiments were conducted for each tested condition.

### Statistical analysis

The mean and standard deviation of each outcome (biovolume, dry-weight and CFU of biofilms, amount of pH drop and hydrogenionic concentration) were calculated for each of the tested conditions. The assumptions of homogeneity of variances and normality of the distribution were checked. Biovolume of *S. mutans* and *C. albicans* on biofilms were individually compared among the distinct sugars by Kruskal-Wallis One-way ANOVA on Ranks followed by Student-Newman-Keuls test. Biovolume of EPS on biofilms formed in the presence of sucrose and starch+sucrose were compared to each other by t-test. Dry-weight and counts of viable cells on biofilms were compared among the distinct sugars by One-way ANOVA followed by Tukey test. Amount of pH drop at 30 minutes interval was compared among the distinct sugar by One-way ANOVA followed by Tukey test, while the amount of pH drop at 4 hours interval and hydrogenionic concentration were compared among the dietary sugars by Kruskal-Wallis One-way ANOVA on Ranks followed by Student-Newman-Keuls test. All analyses were performed on SigmaPlot 12.0 software with a significance level set as 5%.

## Results

### Inter-kingdom co-aggregation in human saliva is sugar-dependent

**Figure 1:**
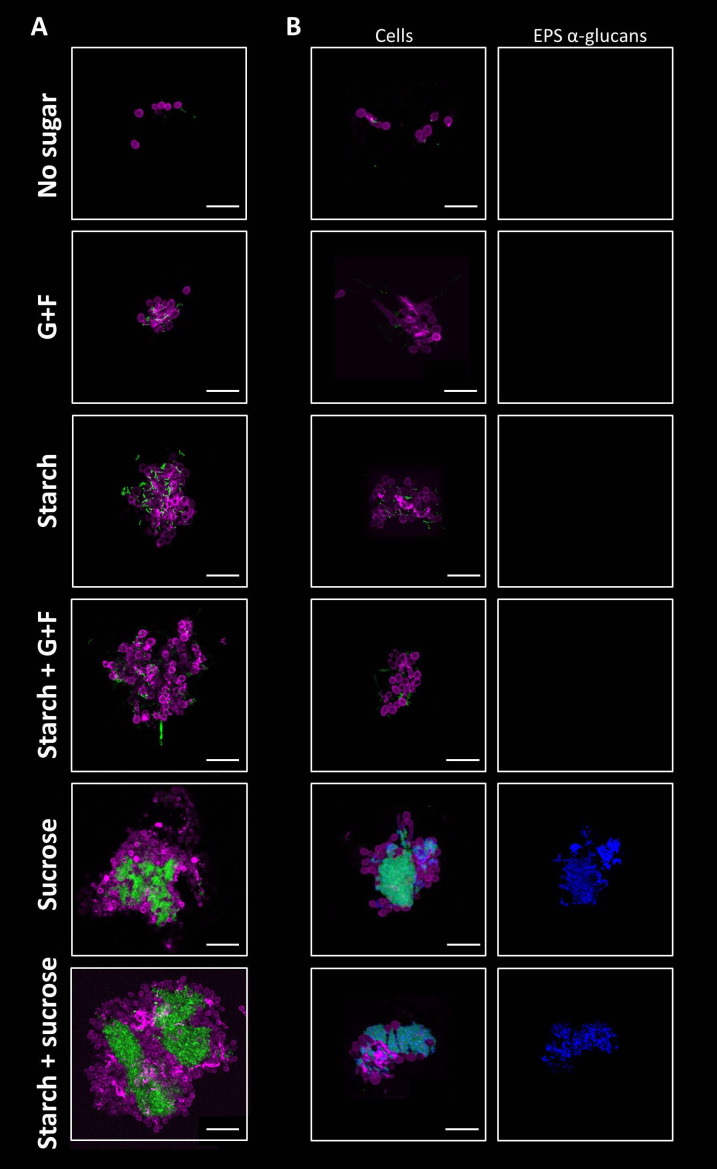
Sugar-mediated inter-kingdom aggregates in human saliva. **(A)** Using fluorescent staining and confocal microscopy, aggregates formed by *S. mutans* and *C. albicans* were found in saliva supplemented with different dietary sugars, which varied in size, structure, and spatial organization depending on the type of sugars. **(B)** EPS α-glucan matrix were found in sucrose-mediated and starch+sucrose-mediated aggregates, but were not detected in aggregates formed in the presence of other dietary sugars, or no sugar. Green, *S. mutans;* purple, *C. albicans*; blue, EPS α-glucans. G+F: glucose+fructose. Scale bar length=25 µm.

To investigate the early interaction between bacteria and fungi in saliva, we first developed a saliva-based experimental model using *C. albicans* and *S. mutans* incubated in clarified human saliva (CLS). We hypothesized that inter-kingdom bacterial-fungal co-aggregation occurs in saliva and this process can be influenced by dietary sugars. To test this possibility, *S. mutans* and *C. albicans* were incubated in CLS without sugar or supplemented with different types of sugars (glucose+fructose, sucrose, starch, or combinations). The microbial structures formed in saliva were fluorescently labelled and analyzed using high-resolution confocal microscopy coupled with computational image analysis. In CLS without sugar, only co-adhered single-cells of *S. mutans* and *C. albicans* was observed after the incubation (**Figure 1A**). In contrast, in the presence of glucose+fructose, starch, or their combination, multicellular aggregates comprised of *S. mutans* chains and *C. albicans* cells (yeasts and pseudo-hyphae) were formed (**Figure 1A**). We also found highly structured inter-kingdom aggregates in CLS supplemented with sucrose or starch+sucrose, wherein densely packed *S. mutans* clusters were co-bound with fungal cells (**Figure 1A**). To understand the assembly mechanism of the inter-kingdom aggregates, we further labelled the EPS glucans. We detected large amounts of α-glucan matrix on the bacterial and fungal cell surfaces within the highly structured aggregates formed with sucrose or starch+sucrose, indicating that α-glucans might contribute to the co-aggregation process (**Figure 1B**). In contrast, α-glucans were not observed within the microbial structures formed in CLS supplemented with glucose+fructose, starch, or their combination (**Figure 1B**), suggesting a glucan-independent co-adhesion mechanism under these conditions. We also quantitatively measured the sizes (surface area in µm^2^) of the inter-kingdom structures using a computational algorithm. Our data revealed different aggregate size distributions among the tested conditions. Small-size microbial structures (< 500 µm^2^) were found in all the tested conditions including CLS with no sugar (**Figure S1A**), whereas middle-size aggregates (500-5,000 µm^2^) were only present in CLS supplemented with dietary sugars (**Figures S1B,C**). Furthermore, we detected large-size aggregates (> 5,000 µm^2^) in CLS supplemented with sucrose and starch+sucrose (**Figure S1D**). These data suggest that dietary sugars, especially sucrose and the combination of starch and sucrose could promote early fungal-bacterial interaction in human saliva by inducing co-aggregation. The inter-kingdom aggregates formed vary in size, structure and spatial organization depending on the type of dietary sugars.

### Sugar-mediated inter-kingdom aggregates display distinctive surface colonization

**Figure 2:**
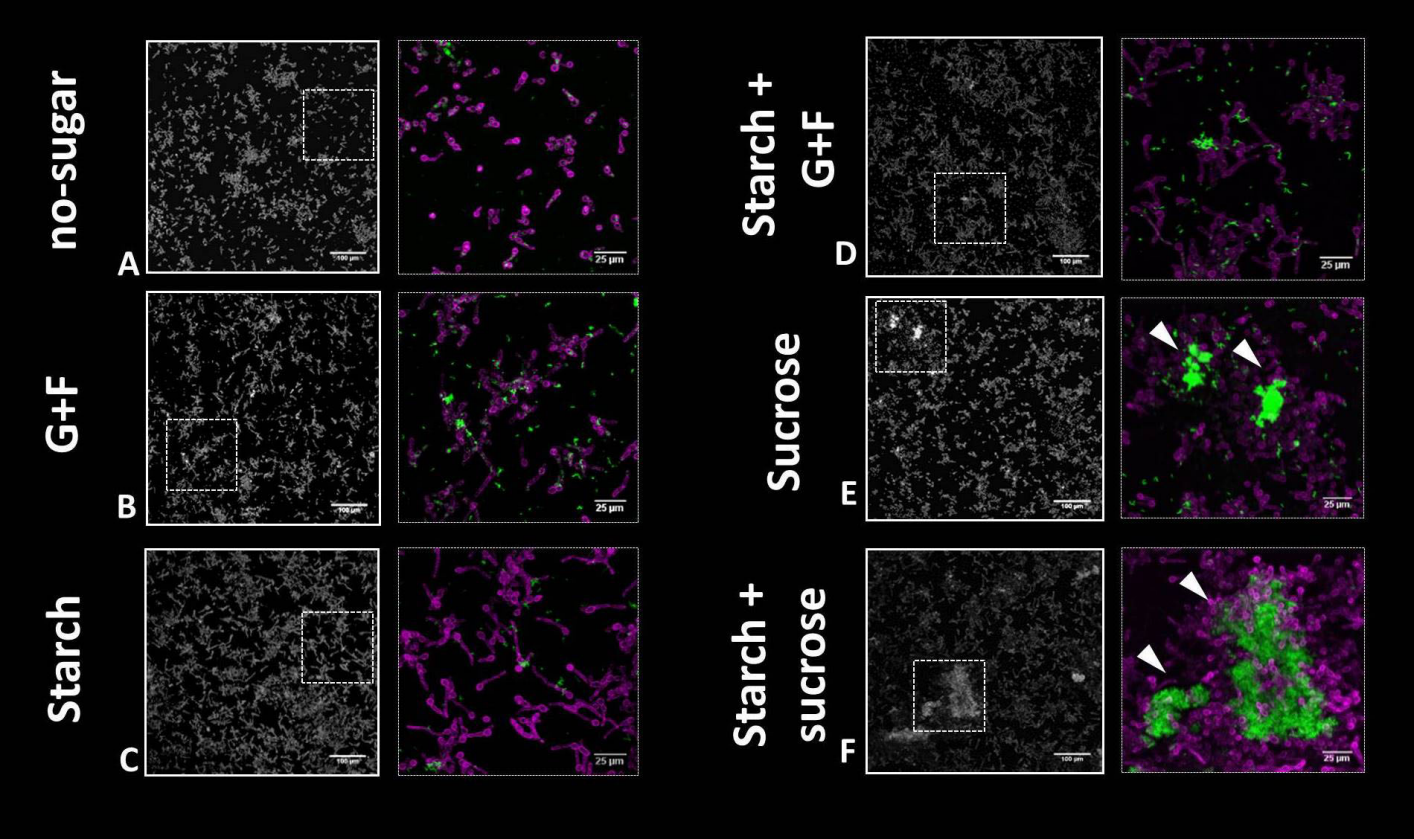
Binding of bacterial and fungal cells onto tooth-mimetic hydroxyapatite surface in saliva supplemented with different dietary sugars. **(A-F)**, representative images (z projection) showing surface-bound bacterial and fungal cells on the surface. The sugar condition is indicated to the left of the images. In each panel, the left image shows a large field of view (640 μm × 640 μm; scale bar = 100 μm) and the right image shows a magnified view (as shown using dotted box in the left image; scale bar = 25 µm). Green, *S. mutans;* purple, *C. albicans*. Arrowheads, sucrose or starch+sucrose-mediated inter-kingdom aggregates attached on the surface. G+F: glucose+fructose.

Given the marked influence of sugars in bacterial-fungal co-aggregation in saliva, we aimed to investigate the binding pattern of microbial structures in saliva onto tooth-mimetic (hydroxyapatite) surfaces. In CLS without sugar, only a sparse colonization of single cells of bacteria and fungi was observed (**Figure 2A**), whereas in the presence of glucose+fructose, starch, or their combination, small aggregates of *S. mutans* chains and *C. albicans* cells (mostly pseudo-hyphae, some yeast cells) were bound on the surface (**Figures 2B-D**). Surprisingly, in addition to small clusters, the large and structured inter-kingdom aggregates formed in CLS supplemented with sucrose or starch+sucrose also attached to the surface as a colonizing unit. These multicellular groups harbor large numbers of *S. mutans* (in densely packed clusters) co-adhered with a network of *C. albicans* yeast and pseudo-hyphae on the surface (**Figures 2E,F**). Together, these findings suggest that dietary sugars could enhance the surface colonization of *S. mutans* and *C. albicans* in saliva. In particular, sucrose and starch+sucrose showed the most pronounced effect, resulting in co-colonization of large numbers of bacteria and fungi onto tooth-mimetic surfaces. The influence of sucrose and starch+sucrose on the subsequent inter-kingdom biofilm development and their disease-causing activities were further investigated.

### Surface-bound inter-kingdom aggregates develop into three-dimensional biofilm structures

**Figure 3:**
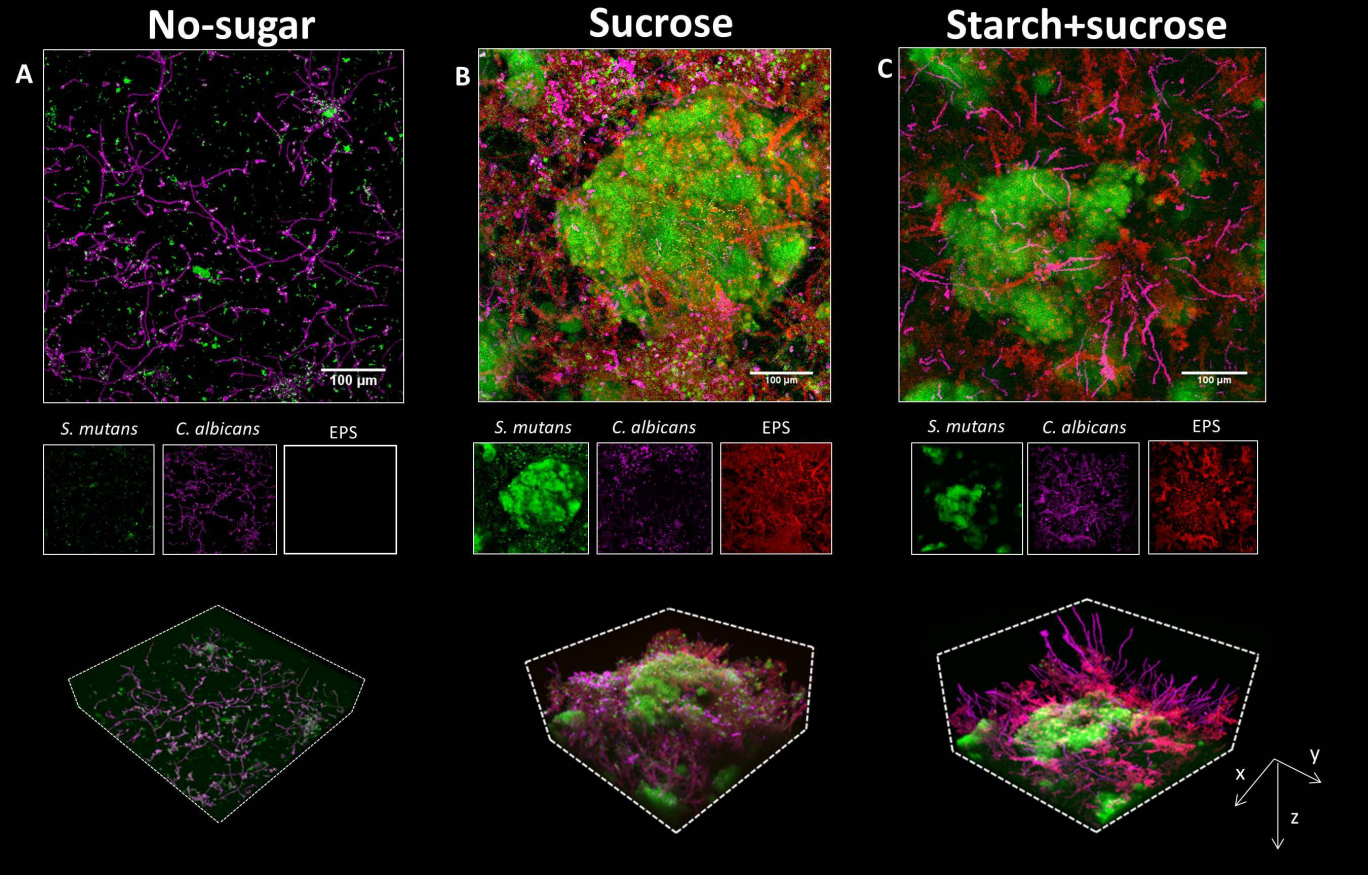
Biofilms formed by surface-bound inter-kingdom aggregates. **(A–C)**, confocal images of the 19h biofilms originated from aggregates mediated by different sugars, as indicated above the images. In each panel, the upper image shows the z projection of a large representative field of view (640 μm × 640 μm; scale bar = 100 μm). The middle images show individual fluorescence channels. The lower images are three-dimensional rendering of the confocal image illustrating the spatial structure of *C. albicans* and *S. mutans*, and EPS matrix. Green, *S. mutans;* purple, *C. albicans;* Red, EPS α-glucans.

Next, we investigated the biofilm formation originated from surface-bound aggregates. After the binding, surface-bound microbes were allowed to grow for 19h in culture medium supplemented with the same sugar used during co-aggregation and surface colonization. The biofilm grown without sugar was thin and sparse, harboring small *S. mutans* clusters and *C. albicans* (mostly in hyphae/pseudo-hyphae forms) (**Figure 3A**, upper panel). Similarly, biofilms formed in the presence of glucose+fructose, starch, and their combinations harbored *S. mutans* clusters and *C. albicans* growing individually on the surface without structural integration (**Figure S2**). In sharp contrast, a complex and highly structured spatial organization was observed in the biofilms formed by sucrose- or starch+sucrose-mediated aggregates, characterized by enlarged *S. mutans* micro-colonies intertwined with *Candida* yeasts, hyphae, and pseudo-hyphae, forming a three-dimensional inter-kingdom superstructure (**Figures 3B, C**, upper panels).

We next investigated how surface-bound aggregates grew spatiotemporally into biofilms using time-lapse live imaging coupled with computational image analysis. We unexpectedly found that the bacterial cells within sugar-mediated inter-kingdom aggregates grew 2.6 times faster than bacteria alone, forming large biofilms (**Figure S3**), suggesting that the structural integration of bacteria and fungi mediated by sucrose may promote their growth dynamics. Individual fluorescence images showed that bacterial and fungal cells within the sucrose- or starch+sucrose-mediated inter-kingdom biofilms were enmeshed by EPS α-glucan, whereas biofilms formed with no sugar or in other sugar-mediated conditions were devoid of EPS (**Figure 3**, middle panels, and **Figure S2**). Notably, the inter-kingdom biofilms formed by starch+sucrose-mediated aggregates appeared to contain more and longer fungal hyphae, protruding outwards from the superstructure (**Figure 3C**, lower panels). Furthermore, orthogonal images showed that biofilms formed by starch+sucrose-mediated aggregates were thicker than those formed by sucrose-mediated aggregates (**Figure S4**). We also performed quantitative image analyses to assess the biovolume of the microbes and EPS. The highest bacterial biovolume was found in the biofilm developed from sucrose-aggregates, followed by those from startch+sucrose-aggregates or other sugars (**Figure S5**). We found significantly more EPS in biofilms formed by starch+sucrose- than sucrose-mediated aggregates (**Figure S6**), suggesting that the combination of starch and sucrose further enhanced EPS production by the inter-kingdom community.

**Figure 4:**
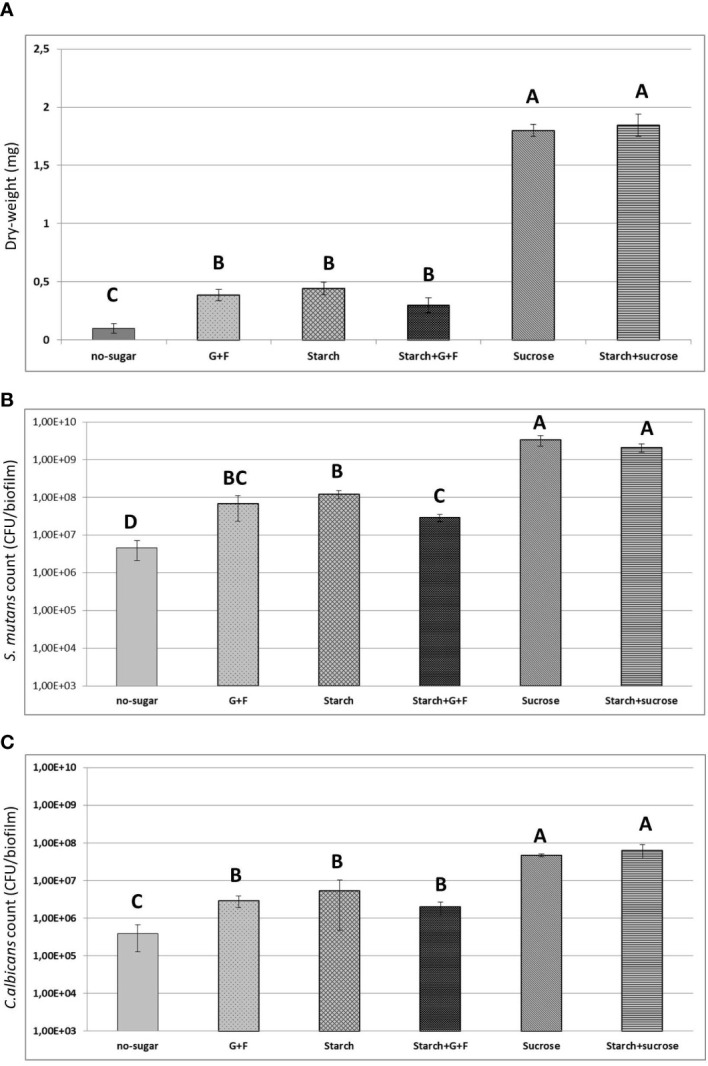
Results of biochemical and microbiological assays. **(A)** Total biomass (measure as dry-weight in mg) of 19h biofilms originated from different sugar-mediated aggregates. **(B)** Counts of *S. mutans* (CFU) within the biofilms **(C)** Counts of *C. albicans* (CFU) within the biofilms. Bars denote the mean and vertical lines denote standard deviation. G+F: glucose+fructose. Groups with distinct capital letter are statistically different (p<0.05 by one-way ANOVA with Tukey test)" by: Groups whose means are followed by different upper case letters differ statistically by ANOVA followed by Tukey test (p<0.05).

We also determined the total biomass (measured as dry-weight) and viable bacterial/fungal cells (measured as CFU) in the biofilms (**Figure 4**). We found over four times more dry-weight in biofilms developed from sucrose- or starch+sucrose-mediated aggregates, compared with biofilms in other conditions (**Figure 4A**). Furthermore, the biofilms from sucrose- or starch+sucrose-mediated aggregates contained at least 10 times more bacteria and fungi (CFU) than those formed in the presence of other sugars. No significant difference (dry-weight or CFU) was found between the sucrose or starch+sucrose group (**Figures 4A–C**). Conversely, the biofilm formed with no sugar had the lowest dry-weight, bacterial and fungal CFU, compared with biofilms developed from sugar-mediated aggregates (**Figure 4**).

Together, our data suggest that the presence of dietary sugars promotes early bacterial-fungal interactions in saliva and co-colonization to the surface as multicellular aggregates. In particular, surface-bound aggregates mediated by sucrose and starch+sucrose develop into enlarged inter-kingdom biofilms harboring elevated amounts of both microbes and EPS spatially arranged into complex three-dimensional superstructures. These striking biomass and structural differences from sugar-mediated aggregates may influence disease-causing virulence of inter-kingdom biofilms.

### Biofilms originated from sucrose and starch+sucrose-mediated aggregates display higher acidogenic potential

**Figure 5:**
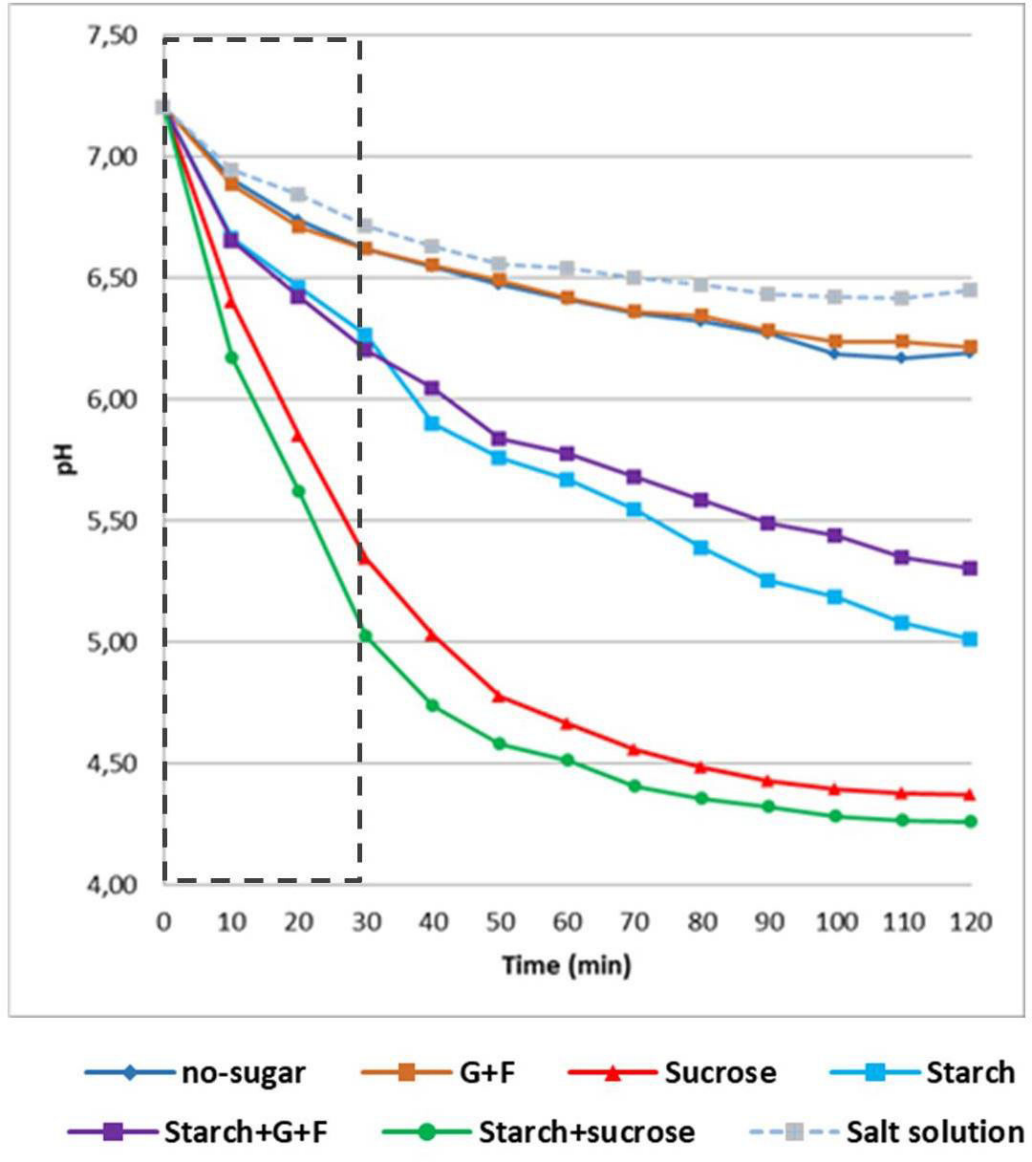
Acidogenic profile of biofilms originated from different sugar-mediated aggregates. Results of glycolytic pH drop assays (glucose-fermenting). 19h biofilms were removed from the culture medium and transferred into a buffer solution containing glucose as the only carbon source. pH of the buffer was measured every 10 min. pH of the fresh culture medium was measured every 2h. Dotted box indicates the data used for proton production analysis (as shown in **Table 1**). G+F: glucose+fructose.

Given the highly structured and densely populated biofilms from sucrose- and startch+sucrose-mediated aggregates, these communities may have higher acidogenic potential. We assessed this possibility using two different assays. We first conducted an established glycolytic pH drop assay (Belli et al., 1995). The data showed that all the biofilms, except those from glucose+fructose-mediated aggregates and the biofilm formed without sugar, were active acid producers and lowered the pH to acidic values (pH < 5.5) (**Figure 5** and **Table 1**), which can cause tooth-enamel demineralization (Stephan, 1944). Intriguingly, the biofilm formed from starch+sucrose-mediated aggregates showed the highest acidogenicity with rapid acid production, followed by the biofilm from sucrose-mediated aggregates, reaching highly acidic pH values (final pH 4.2-4.4). This finding was corroborated by the second assay, in which the same biofilm was transferred into fresh culture medium supplemented with the same dietary sugars and allowed to further grow overnight. We found that the biofilm from sucrose- and starch+sucrose-mediated aggregates were the most acidogenic (final pH ~ 4.6), followed by biofilms from other sugar-mediated aggregates (**Figure S7** and **Table S1**). In both assays, biofilms formed without sugar were the least acidogenic maintaining pH values around 6.5.

**Table 1:** Acidogenic potential of biofilms (means ± standard deviation) over 30 min of fermentation of glucose.

Variables	No sugar	G+F	Sucrose	Starch	Starch+G+F	Starch+sucrose
Amount of pH drop/min	0.019 ± 0.001^d^	0.019 ± 0.001^d^	0.062 ± 0.006^b^	0.031 ± 0.004^c^	0.033 ± 0.004^c^	0.073 ± 0.002^a^
Hydrogenionic concentration (µmol/L)	0.614 ± 0.026^C^	0.633 ± 0.014^C^	6.707 ± 2.434^B^	1.190 ± 0.164^BC^	1.316 ± 0.274^BC^	12.883 ± 2.336^A^

Means followed by different lower case superscript letters differ statistically by ANOVA followed by Tukey test (p<0.05). Means followed by different upper case superscript letters differ statistically by Kruskal-Wallis ANOVA on Ranks followed by Student-Newman-Keuls test (p<0.05). Salt solution: 0.016 ± 0.002 (pH drop/min) and 0.52 ± 0.08 (H^+^ concentration; µmol/L) G+F: glucose+fructose.

Collectively, our data show distinctive acidogenicity profiles of biofilms formed by different sugar-mediated aggregates. Among them, biofilms originated from starch+sucrose- and sucrose-mediated aggregates formed inter-kingdom biofilms that harbors dense populations of both bacteria and fungi which are highly structured and acidogenic, indicating that these sugars in saliva may contribute to the enhanced biofilms virulence in childhood caries.

## Discussion

Our findings reveal that dietary sugars can affect early bacterial-fungal interactions in human saliva to modulate inter-kingdom biofilm development on the surface. We demonstrate three key features: (i) Dietary sugars influence co-aggregation between *S. mutans* and *C. albicans* in human saliva. These aggregates vary in size, structure, and spatial organization depending on the type of sugars. (ii) Sucrose and starch in combination with sucrose induce aggregates that are larger and highly structured, characterized by bacterial clusters co-bound with fungal cells enmeshed by an extracellular glucan matrix. (iii) These sugar-mediated aggregates can attach to the tooth-mimetic surface and grow into highly acidogenic biofilms with increased biomass and cohesion, whereas biofilms derived without sugars display lack of structural integrity and reduced acidogenicity. The data suggest complex and multifaceted mechanisms governing the assembly of inter-kingdom aggregates involving cell-cell, cell-EPS, and cell-saliva interactions which are influenced by dietary sugars. *S. mutans* and *C. albicans* can bind to each other *via* streptococcal cell surface proteins (antigens I/II) and cell wall adhesins of *C. albicans* (e.g., agglutinin-like sequence) (Silverman et al., 2010; Xu et al., 2014; Yang et al., 2018; Negrini et al., 2019). Conversely, salivary proteins, such as proline-rich proteins adsorbed onto oral streptococci surface may act as receptors for *C. albicans* adhesion (O´Sullivan et al., 2000). *C. albicans* is also able to adsorb salivary mucin (Edgerton et al., 1993) which may facilitate co-adhesion. However, in the presence of sucrose, with or without starch, the binding interactions changes dramatically partially due to exoglucans production by *S. mutans* glucosyltransferases (Gtfs) (Gregoire et al., 2011; Falsetta et al., 2014; Hwang et al., 2015).

Sucrose can enhance bacterial-fungal interactions in at least two interconnected ways: Gtf binding on the fungal surface and *in situ* EPS-glucan formation. The interaction between *S. mutans* and *C. albicans* can be mediated by the *S. mutans*-derived Gtf exoenzymes, which bind avidly onto the mannan layer of *Candida* cell-wall surface in active form, converting sucrose to EPS α-glucans *in situ* (Hwang et al., 2017). The EPS glucans formed on the fungal surfaces provide bacterial binding sites for *S. mutans* through cell membrane-associated glucan binding proteins (GBPs) (Banas and Vickerman, 2003; Bowen and Koo, 2011). The binding force between *S. mutans* and glucan-coated *C. albicans* is 6-fold stronger than that on the uncoated fungal surface (Wan et al., 2021), leading to a cohesive and stable inter-kingdom co-binding. The presence of starch could have an additional effect on co-aggregation. Salivary alpha-amylase may contribute to bacterial-fungal co-aggregation by converting starch into starch hydrolysates, which act as glucans precursors during the EPS biosynthesis (Bowen and Koo, 2011). Amylase also binds to Gtf on *S. mutans* surfaces in active form while enhancing Gtf activity (Chaudhuri et al., 2007), which may further promote glucan synthesis *in situ* and co-binding interactions. Although direct cell-to-cell interactions can occur, EPS glucans appears to play a dominant role in the inter-kingdom co-aggregation in saliva when sucrose (or with starch) is present.

Interestingly, the large inter-kingdom aggregates formed in sucrose or sucrose with starch can bind the apatitic surface as colonizing units. Previous studies have shown that exoglucans can facilitate the binding of single-cells of *S. mutans* and *C. albicans* to saliva-coated apatitic surfaces (Schilling and Bowen, 1992; Gregoire et al., 2011). In addition, Gtfs and alpha-amylase are also found adsorbed onto the salivary pellicle in active forms, converting sucrose and starch hydrolysates (oligosaccharides) into EPS glucans (Vacca-Smith et al., 1996; Duarte et al., 2008) that can enhance microbial surface colonization. Therefore, it is possible that the inter-kingdom aggregates containing exoglucans have higher binding affinity to the surface, while the *in situ* EPS synthesis by Gtf on salivary pellicle can further strengthen the binding and promote accumulation of microbes. Moreover, in the presence of starch and sucrose, the combined enzymatic activity of surface-adsorbed amylase and Gtf produce EPS with more insoluble glucans (Vacca-Smith et al., 1996) that can further enhance microbial colonization. These properties together may explain the surface colonization of large inter-kingdom aggregates and its further accumulation in the presence of sucrose or sucrose combined with starch. Conversely, in the absence of these sugars, *S. mutans* and *C. albicans* adhere to saliva-coated apatite surface in an EPS-independent manner with fewer cells and smaller clusters likely through cell surface adhesins and salivary pellicle receptors (Whittaker et al., 1996; Kolenbrander et al., 2010).

The sucrose-mediated (either alone or combined with starch) aggregates led to enhanced biofilm formation, whereby large and densely packed *S. mutans* cells were intertwined with numerous fungal cells. This enhancement could be attributed to the symbiotic relationship between *S. mutans* and *C. albicans* when they are growing together within biofilms, including complex cross-feeding and metabolic exchanges that promoted mutual growth (Falsetta et al., 2014; Sztajer et al., 2014; Kim et al., 2017). Although *S. mutans* can utilize various dietary sugars as carbon sources, *C. albicans* does not efficiently metabolize sucrose in the conditions tested here but can readily utilize fructose and glucose (Sztajer et al., 2014; Kim et al., 2017; Ellepola et al., 2019). *S. mutans*-derived Gtf exoenzymes convert sucrose to glucose that can be more readily metabolized by *C. albicans* to enhance fungal growth and acid production (Sztajer et al., 2014; Kim et al., 2017). In addition, the presence of *C. albicans* in the mixed biofilm can increase the amount of EPS by inducing *S. mutans*-derived *gtf* expression as well as by direct contribution *via* production of its own exopolymers (β-glucans) (Falsetta et al., 2014). Furthermore, biofilms formed in the presence of starch and sucrose contain a higher amount of 3-linked glucan branching (3,4-, 3,6-, and 3,4,6-linked glucose) compared to those grown in sucrose alone (Duarte et al., 2008). These altered glucans are more insoluble and contribute to biofilm scaffolding, which may explain the increased EPS biovolume found in the inter-kingdom aggregate-mediated biofilm formed in the presence of sucrose and starch.

The accumulation of biofilms with denser microbial population and increased amounts of EPS can augment its acidogenicity, a key hallmark in caries pathogenesis. The enhanced colonization and carriage of both microorganisms can amplify acid production of the inter-kingdom biofilms *via* cross-feeding and sucrose breakdown (Sztajer et al., 2014; Kim et al., 2017). The presence of thicker and well-developed EPS matrix can create diffusion-limiting barriers that promote localized acidogenesis and acidic microenvironment (Xiao et al., 2012; Hwang et al., 2016; Bowen et al., 2018). Glucans can increase biofilm porosity, modulating the diffusion of small molecules and monosaccharides throughout the biofilm thickness allowing faster pH drops at tooth/biofilm interface (Bowen and Koo, 2011). Notably, glucans can also trap protons further enhancing acidification within biofilm (Guo et al., 2015). Moreover, the extracellular glucans can also serve as a carbohydrate source that allows prolonged acid production during starvation. Our findings that biofilms originated from inter-kingdom aggregates display the highest acid production rates further confirms the acidogenic interactions, but also provide new insights on how sucrose and starch promote co-existence early on in saliva boosting bacterial-fungal symbiosis on the apatitic surface. This indicates that dietary sugars may have a cariogenic role even before microbial colonization, which can influence both the accumulation and acidogenicity of biofilms. How dietary sugars modulate the dynamics of inter-kingdom biofilm development and how metabolic exchanges are orchestrated in the presence of different sugar remain understudied. Further studies are needed to investigate the spatiotemporal dynamics during the inter-kingdom biofilm development.

## Conclusion

In summary, we find diverse bacterial-fungal aggregates in saliva which vary in size, structure, and spatial organization depending on the type of sugars. Notably, both colonization and further growth on the apatitic surface were dependent on sugar-mediated aggregation, leading to biofilms with distinctive spatial organizations and 3D architectures. Sucrose and sucrose with starch induce the formation of large inter-kingdom aggregates that colonize, actively grow, and develop into thick biofilms harboring a complex network of bacterial and fungal cells (yeasts and hyphae) surrounded by an abundant EPS matrix that are highly acidogenic, lowering the pH values to cariogenic levels. In contrast, biofilms originated from microbial cells in saliva with other sugars (or without sugar) were sparsely bound on the apatitic surface without structural integration, growing predominantly as individual species with reduced acidogenicity. These findings reveal the impact of dietary sugars on inter-kingdom interactions in saliva and how they influence cariogenic potential even before microbial colonization. Specifically, sucrose and starch can modulate inter-kingdom interactions earlier than previously considered by promoting co-assembly and structural organization in saliva that ultimately facilitates surface colonization and cariogenic biofilm formation. This conceptual framework suggests that early therapeutic strategies could disrupt these inter-kingdom aggregates in saliva to prevent the initiation of pathogenic biofilms implicated with severe childhood caries.

## Data availability statement

The raw data supporting the conclusions of this article will be made available by the authors, without undue reservation.

## Ethics statement

This study was reviewed and approved by the Institutional Review Board of University of Pennsylvania (IRB #824243). The saliva donors provided their written informed consent to participate in this study. 

## Author contributions

TN, ZR, ÁS-S, HK, and RA contributed to conception and design. TN and RA performed the experiments. TN, YM, DK, YL, and RA acquired data. TN, HK, and RA contributed to data analysis and data interpretation. TN, ZR, HK, and RA drafted the manuscript. All authors contributed to the article and approved the submitted version.

## Funding

This work was supported by the National Institute for Dental and Craniofacial Research Grant DE025220 (to HK). ZR is supported by the NIDCR Postdoctoral Training Program under Award Number R90DE031532. The content is solely the responsibility of the authors and does not necessarily represent the official views of the funders.

## Acknowledgments

The authors would like to thank Coordenação de Aperfeiçoamento de Pessoal de Nível Superior [CAPES-PEVEX 88881.171614/2018-01 (RA)] and Fundação de Amparo à Pesquisa do Estado de São Paulo [FAPESP-BEPE 2018/18258-8 (TN)]. The authors would like to thank Dr. Geelsu Hwang for his supervision on the calculation of the surface area covered by bacterial-fungal aggregates.

## Conflict of interest

The authors declare that the research was conducted in the absence of any commercial or financial relationships that could be construed as a potential conflict of interest.

## Publisher’s note

All claims expressed in this article are solely those of the authors and do not necessarily represent those of their affiliated organizations, or those of the publisher, the editors and the reviewers. Any product that may be evaluated in this article, or claim that may be made by its manufacturer, is not guaranteed or endorsed by the publisher.
