# Supplementary material for: Dietary sugars modulate bacterial-fungal interactions in saliva and inter-kingdom biofilm formation on apatitic surface
Source: Front Cell Infect Microbiol. 2022 Nov 9;12:993640. doi: 10.3389/fcimb.2022.993640 (PMC9681999; doi:10.3389/fcimb.2022.993640)
Supplement: Supplementary file 1 [file DataSheet_1.pdf]

Supplementary Material

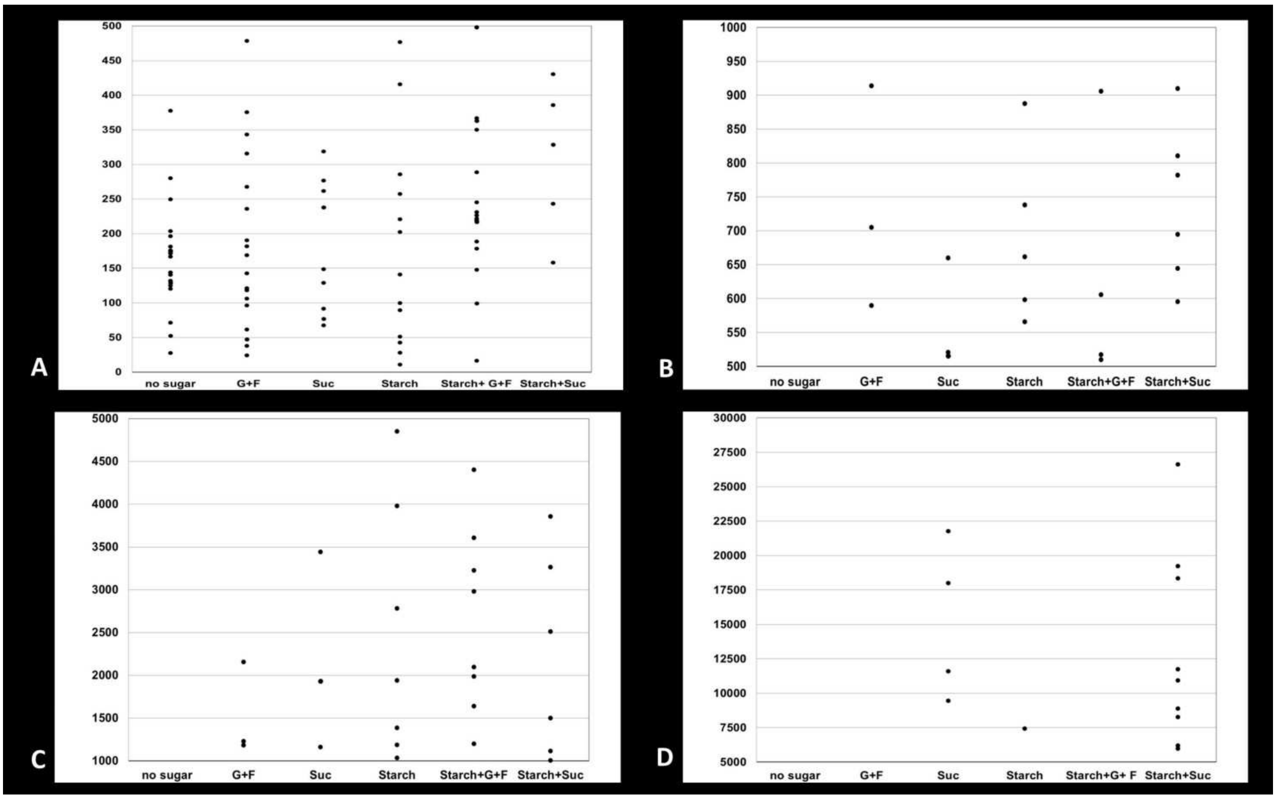

Figure S1. Size distribution (surface area; µm^2) of inter-kingdom aggregates mediated by different dietary sugars. (**A**) Small-sized microbial structures (0 to 500 µm^2). **(B)** Middle-sized aggregates (lower range, 500 to 1,000 µm^2). **(C)** Middle-sized aggregates (higher range, 1,000 to 5,000 µm^2). **(D)** Large-sized aggregates (5,000 to 30,000 µm^2). G+F: glucose+fructose; Suc: sucrose.

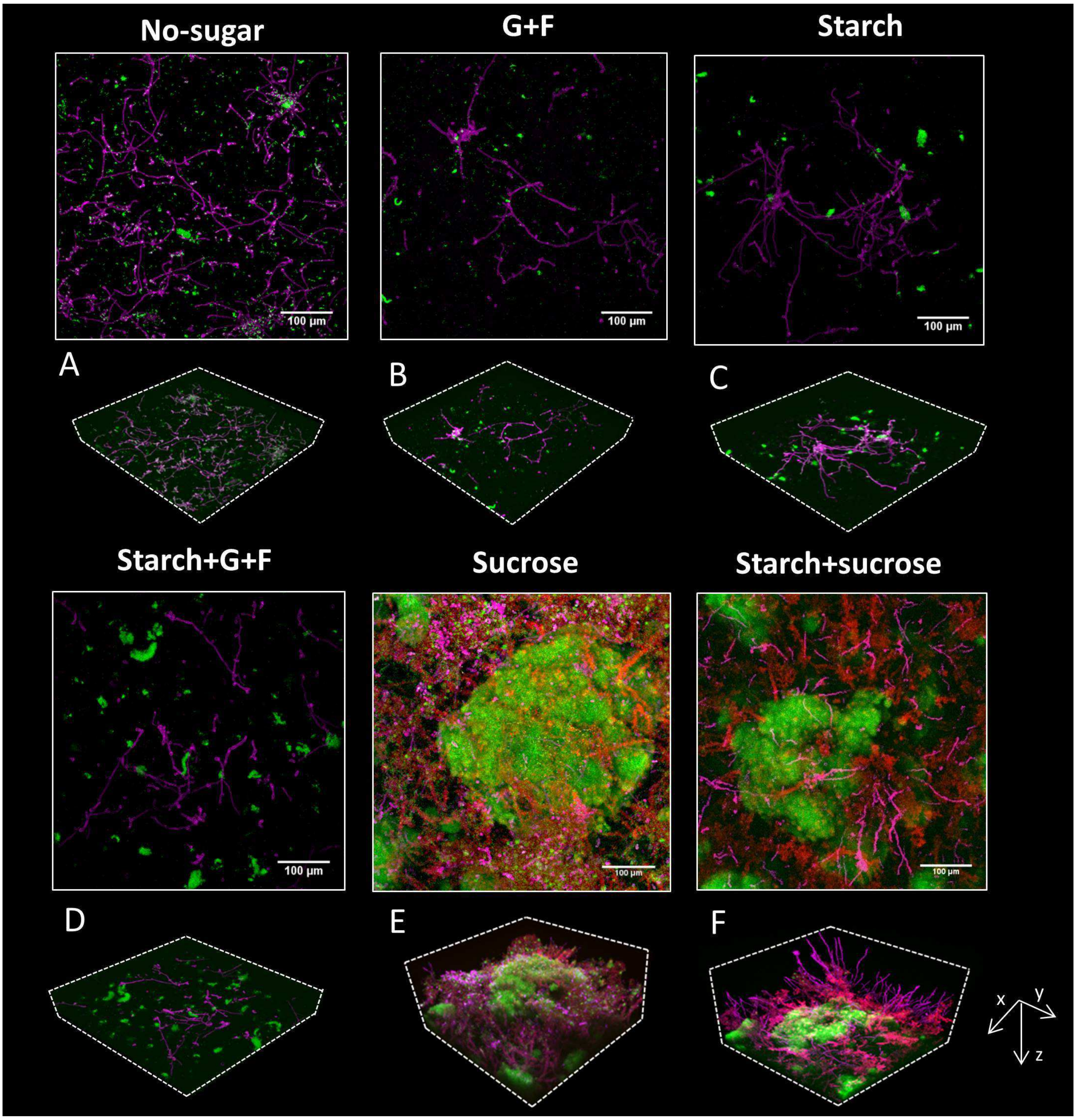

Figure S2. Confocal images of the 19h biofilms originated from aggregates mediated by different sugars, as indicated above the images. (**A**) non-sugar; (**B**) Glucose+fructose (G+F); (**C**) Starch; **(D)** Starch+G+F; (**E**) Sucrose; (**F**) Starch+sucrose. In each panel, the upper image is a z projection and the lower image is a three-dimensional rendering of the confocal data. Green, *S. mutans;* purple, *C. albicans;* Red, EPS α-glucans.

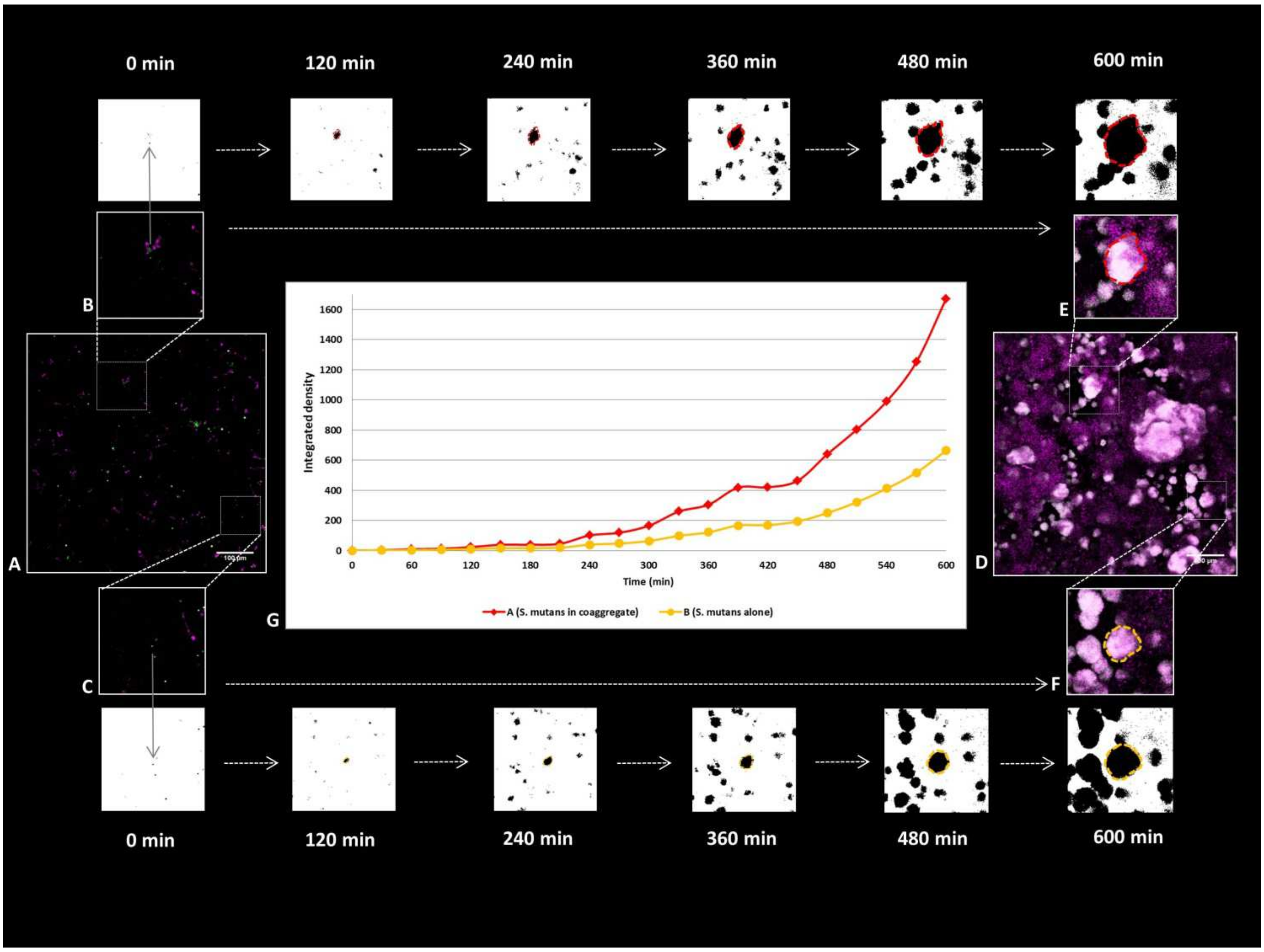

Figure S3. Growth dynamics of surface-bound interkingdom aggregates. (**A**) Confocal image (z projection) of a representative field of view (640 µm × 640 µm) with surface-attached microbial structures mediated by sucrose at 0 min. Both bacterial-fungal aggregates and isolated bacterial aggregates are present within the initial attached community. (**B**) Region of interest (ROI) containing a fungal-bacterial aggregate attached at 0 min. The image series above panel B shows the bacterial channel (segmented) of the same ROI over time, and the red outline indicates the area of the bacteria inside the growing aggregate, which was followed and computationally quantified as detailed in the Materials and Methods (**C**) ROI containing an isolated bacterial aggregate at 0 min. The image series below panel C shows the bacterial channel (segmented) of the same ROI over time, and the red outline indicates the area of the growing bacteria, which was also followed and computationally quantified. **(D)** The same field of view as in A at 600 min. (**E**) Image of the same ROI as in B at 600 min. (**F**) Image of the same ROI as in C at 600 min. (**G**) Time-resolved Raw Integrated Density of bacteria within the inter-kingdom aggregate (curve in red) and the isolated bacteria alone (curve in yellow).

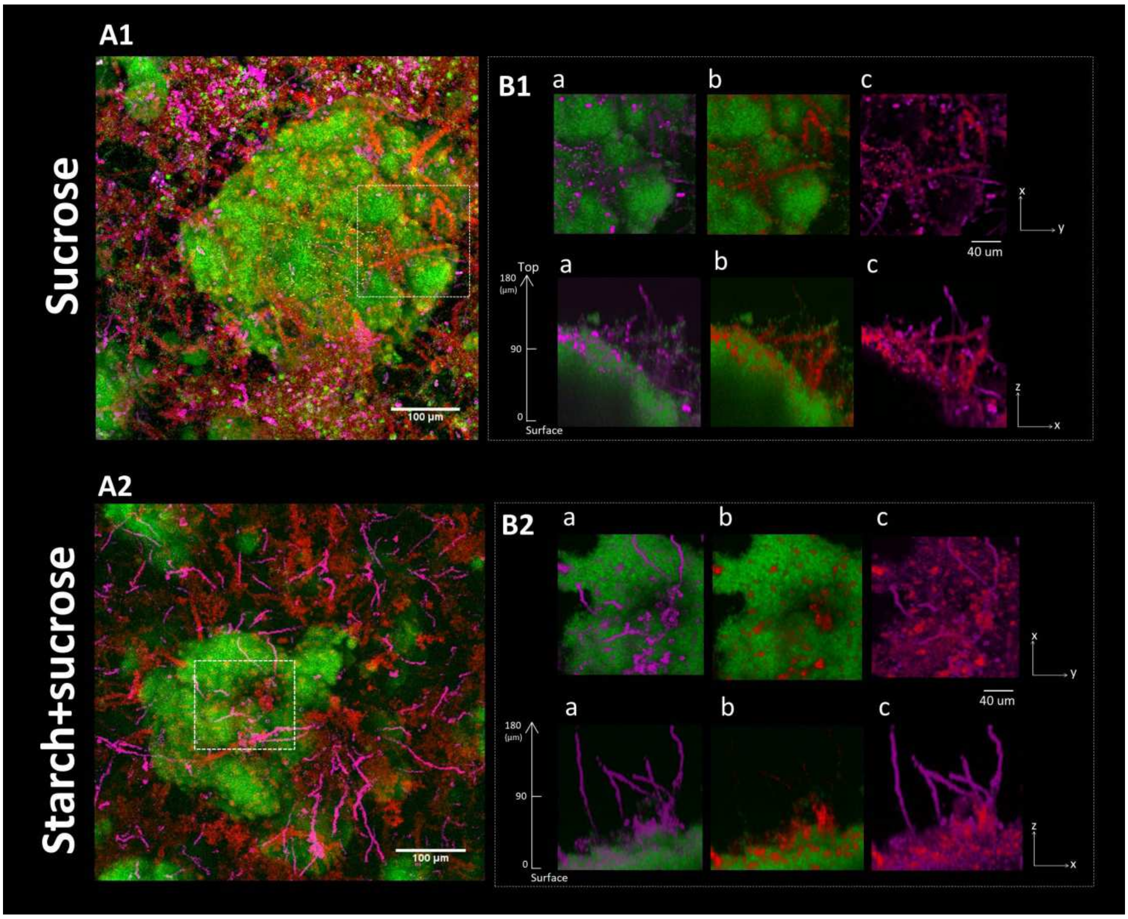

Figure S4. Confocal fluorescence image (z projection and orthogonal projection) of the biofilms originated from sucrose or starch+sucrose-mediated aggregates. (**A1, A2**) 19h biofilm formed by sucrose-mediated aggregates or starch+sucrose-mediated aggregates, respectively. Dotted box, selected area analyzed using z projection and orthogonal projection. (**B1, B2**) top, z projection of the analyzed area showing intertwined *S. mutans* micro-colonies and *C. albicans*, surrounded by an intricate matrix of extracellular α-glucans. bottom, orthogonal projection of the analyzed area showing the thickness of the biofilm superstructure. Green, *S. mutans;* purple, *C. albicans;* red, EPS α-glucans. In B1 and B2, **a**, merged image of *S. mutans* and *C. albicans*; **b**, merged image of *S. mutans* and EPS; **c**, merged image of *C. albicans* and EPS.

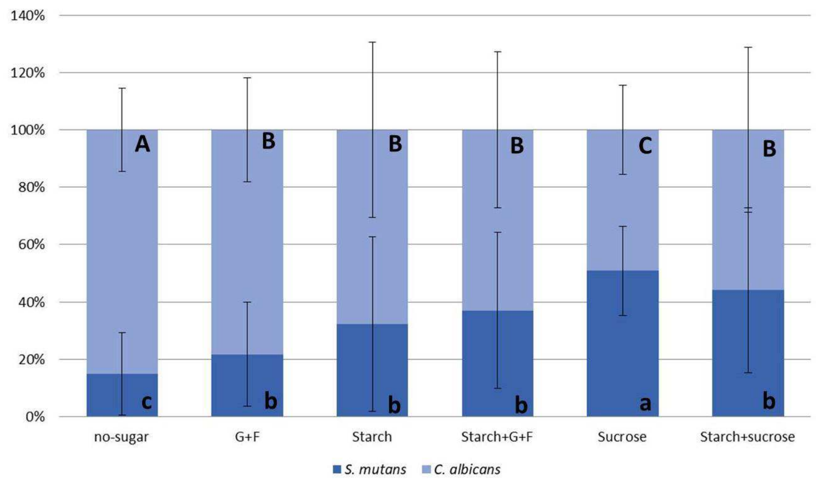

Figure S5. Quantitative analysis of biovolume of *S. mutans* and *C. albicans* in biofilms originated from different sugar-mediated aggregates. Data are presented as percentage of each species' biovolume in the total biofilm volume (% of total biovolume). Bars denote mean and vertical bars denote standard deviation. Distinct lower-case letters and upper-case letters indicate statistical difference on *S. mutans* and on *C. albicans* biovolume, respectively. ($p < 0.05$, Kruskal-Wallis One-way ANOVA on Ranks followed by Student-Newman-Keuls test). G+F: glucose+fructose.

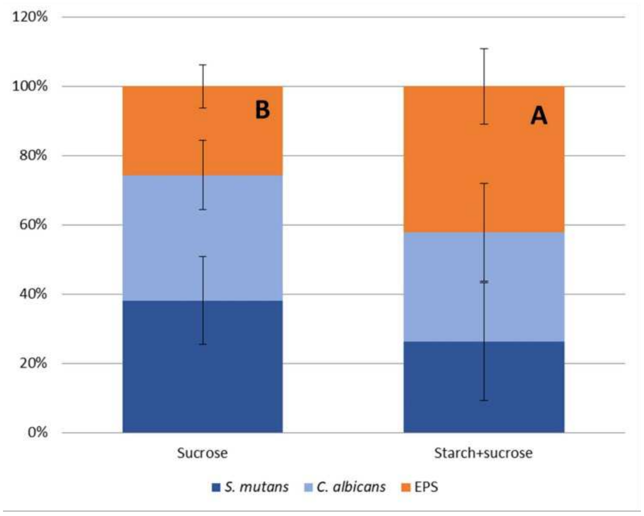

Figure S6. Quantitative analysis of biovolume of EPS in biofilms originated from sucrose-mediated or startch+sucrose-mediated aggregates. Data are presented as percentage of the EPS biovolume in the total biofilm volume (% of total biovolume). Bars denote mean and vertical bars denote standard deviation. Distinct upper-case letters indicate statistical difference on EPS biovolume ($p < 0.05$, t-test).

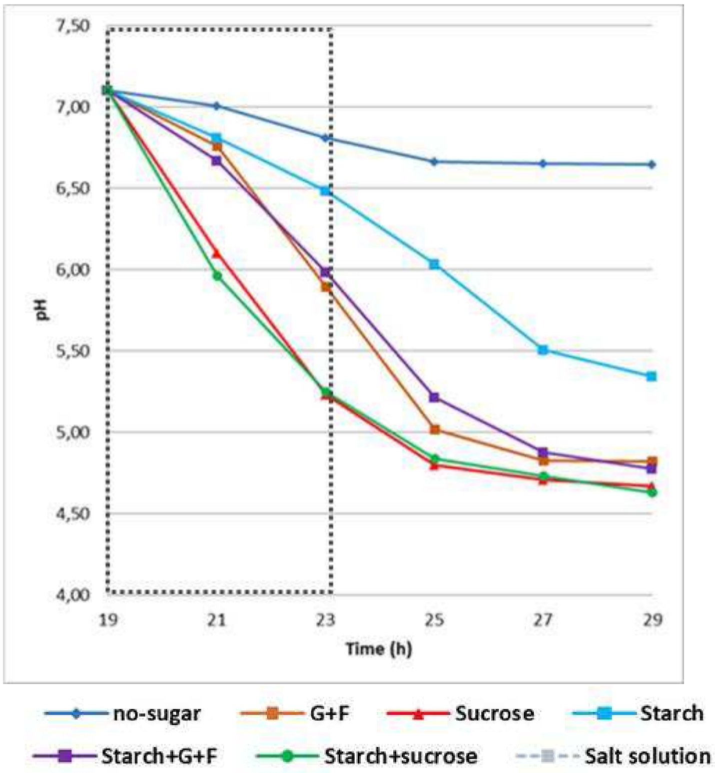

Figure S7. Acidogenic profile of biofilms originated from different sugar-mediated aggregates. pH of the culture medium over time. 19h biofilms were transferred into fresh culture medium supplemented with the same dietary sugar (or combinations) used during the co-aggregation, binding, and biofilm formation. pH of the fresh culture medium was measured every 2 h. Dotted box indicates the data used for proton production analysis (as shown in Table S1)

Table S1. Acidogenic potential of biofilms (means ± standard deviation) over 4h of fermentation of the dietary sugars.

Variables	No-sugar	G+F	Sucrose	Starch	Starch+G+F	Starch+sucrose
Amount of pH drop/h	0.073 ± 0.007^{D}	0.301 ± 0.015^{A}	0.467 ± 0.007^{A}	0.154 ± 0.011^{C}	0.279 ± 0.004^{B}	0.463 ± 0.006^{A}
Hydrogenionic concentration (µmol/L)	0.334 ±0.010^{C}	1.535 ± 0.167^{A}	6.775 ± 0.498^{A}	0.565 ± 0.050^{B}	1.336 ± 0.048^{A}	6.844 ± 0.363^{A}

Means followed by different upper case superscript letters differ statistically by Kruskal-Wallis ANOVA on Ranks followed by Student-Newman-Keuls test ($p<0.05$).
